# Melatonin and Sleep-Wake Rhythms before and after Ocular Lens Replacement in Elderly Humans

**DOI:** 10.3390/biology5010012

**Published:** 2016-02-15

**Authors:** Marina Giménez, Domien Beersma, Serge Daan, Bert van der Pol, Martijn Kanis, Dick van Norren, Marijke Gordijn

**Affiliations:** 1 Department of Chronobiology, GeLifes, University of Groningen, Groningen 9747 AG, The Netherlands; d.g.m.beersma@rug.nl (D.B.); s.daan@rug.nl (S.D.); m.c.m.gordijn@rug.nl (M.G.); 2 Chrono@Work B.V., Groningen 9747 AT, The Netherlands; 3 Department of Ophthalmology, University Medical Centre Groningen, Groningen 9713 GZ, The Netherlands; vdpol@me.com; 4 Department of Ophthalmology, University Medical Centre Utrecht, Utrecht 3584 CX, The Netherlands; mkanis@tergooi.nl (M.K.); d.vannorren@gmail.com (D.N.)

**Keywords:** aging, ocular lens, cataract surgery, sleep-wake rhythms, melatonin rhythms, humans

## Abstract

Light of short wavelengths has been shown to play a key role in non-image forming responses. Due to aging, the ocular lens becomes more yellow reducing the transmission of short wavelengths in the elderly. In the present study, we make use of cataract surgery to investigate the effects of a relative increase of short wavelength transmission on melatonin- and sleep-wake rhythms (N = 14). We observed, on average, a delay of the sleep-wake and the nocturnal melatonin rhythms after cataract surgery. This delay is tentatively attributed to a relatively large increase of light transmittance in the evening hours more than an increase of the already relatively high light intensities found in the daytime. The later phase that we observed after cataract surgery (clear lens) as compared to the earlier phase observed before cataract (yellowish lens) is in agreement with the general later phase reported in the young (clear lens) population.

## 1. Introduction

Humans display a multitude of circadian rhythms in physiology and behavior, each with their own specific timing with respect to day and night. Many of these rhythms are under (partial) control of a circadian pacemaker located in the suprachiasmatic nuclei (SCN) of the hypothalamus. The SCN in turn is entrained to the systematic 24-h variations of the environment. In humans, this is almost exclusively achieved by daily adjustments through exposure to the light-dark cycle. The spectral composition of light has been shown to be critical for these so-called non-image forming (NIF) responses to light. Short wavelengths (circa 460–480 nm) in particular are of importance for inducing these responses [1,2,3,4,5,6]. This is related to the role of the photoreceptive retinal ganglion cells (pRGCs) containing the blue-sensitive pigment melanopsin. pRGCs connect directly with SCN neurons [7,8,9,10] and other regions such as the ventrolateral preoptic area (VLPO), a key region for sleep regulation [11].

There are some indications that in the elderly population the overt rhythms generated by the circadian system are disturbed. This is shown, although not consistently across studies, for instance in fragmented sleep-wake rhythms, lower amplitude of melatonin rhythms as well as in the reduced amplitude in rhythms already present at the level of the SCN such as vasopressin (for review see [12]). Also, when compared to young subjects, reports have shown that the sensitivity of the circadian system of the elderly to phase shifting is different depending on light intensity [13] and timing of the light exposure [14], although the maximum response may remain similar [13]. When compared to young participants, elderly subjects also show a different phase angle relationship between melatonin and sleep-wake rhythms [15]. This difference may to some extent explain the disrupted sleep that is usually reported in the elderly as compared to the young. As a matter of fact, phase angle differences can be used as a marker for circadian alignment and be used as a tool for diagnosing circadian phase disorders [16]. It is conceivable that this reduction in the strength of circadian expression is partly related to reduced input of light. In particular, short wavelengths are filtered out as a consequence of a progressive yellowing of the ocular lens with age [17]. Although it is a tempting hypothesis, there is no clear evidence for the idea that reduced circadian rhythmicity is indeed the result of such a reduction in light input due to lens transmittance. Moreover, in elderly organisms, not only the ocular lens becomes denser but also structural changes at the level of the retina occur [18,19,20] that may mediate to some extent the amount of light available for the NIF system. It is also not yet fully determined whether replacement of the aged lenses by artificial ones after cataract surgery would partly reverse the effects. In The Netherlands, cataract surgery is conducted in large numbers (in the order of 100,000 lens replacements per year). In this study, we exploit cataract surgery to investigate the effects of removing the age-related reduction of short wavelength transmission through the ocular lens on melatonin and sleep-wake rhythms. If the observed changes in robustness of the circadian system are mainly due to the age-related changes of the ocular lens, the implantation of a new transparent lens is expected to increase the Zeitgeber strength and thereby affect the entrained phase of sleep-wake and melatonin rhythms as well as restore their robustness. These hypotheses are tested in the present study.

## 2. Methods

### 2.1. Subjects

Subjects were recruited from the waiting list for cataract surgery on both eyes at the University Medical Center in Groningen (UMCG), The Netherlands. Subjects had to be older than 65 years and free of medical conditions. Subjects completed a general health questionnaire. Sleep characteristics were not used as a criterion for inclusion or exclusion. Those who had been traveling on transmeridian flights within a month before the initiation of the study protocol were excluded. All subjects gave written informed consent and were paid for participation. The experimental protocol was approved by the Medical Ethics Committee of the UMCG. The study took place between April 2007 and February 2009.

### 2.2. Experimental Design

Measurements were conducted before and after surgery. The first assessment started between one and two months before the replacement of the first eye lens. The operation on the second eye occurred maximally two months after the operation of the first eye. The second assessment was conducted at least four weeks (on average 3.5 ± 3 months) after the operation on the second eye to allow for recovery.

The following measurements were made before and after bilateral lens replacement:

Actigraphy: During three consecutive weeks actigraphy data were collected (Actiwatch^®^, Cambridge Neurotechnologies, Cambridge, UK). Movements of the non-dominant arm were recorded per 1-min bin. During those three weeks, sleep-logs (going to bed and waking up times) were completed on a daily basis.

Melatonin profile: During the same three weeks, subjects came to the lab for one night to assess their salivary melatonin profile. In three cases, subjects collected the saliva samples at home, either with strict instructions (*n* = 2) or under supervision (*n* = 1). In all cases, subjects stayed under dim light conditions (< 5 lux) from 17:00 h local time at the date of collection, *i.e.*, Central European Time (CET) in winter, and Daylight Saving Time (DST) = CET + 1 in summer. Sleep started at their usual bedtime. During sleep, all lights were off. Saliva samples were collected once per hour from 18:00 h till 1:00 h and once per two hours from 1:00 h till 9:00 h. Food intake was restricted to the first 15 min after each sample. Chocolate, bananas, coffee and tea were not allowed during the whole sampling period. Ten minutes prior to each sample, subjects were asked to sit quietly to avoid influence of posture [21]. Saliva samples were centrifuged immediately after collection and stored at −20 °C until analysis when samples were collected in the lab, or on the next day when samples were collected at subjects’ home. Melatonin concentration was assessed by radio-immunoassay (RK-DSM, Bühlmann laboratories AG, Alere Health B.V., Tilburg, The Netherlands). All samples from the same individual were analyzed within the same series. The limit of detection for the RIA was 0.3 pg/mL with an intra-assay variation of 6.7% at a low melatonin concentration (mean 1.5 pg/mL, *n* = 30) and 6.5% at a high melatonin concentration (mean = 15 pg/mL, *n* = 30). Inter-assay variation was 12.2% at low melatonin concentration (mean = 2.1 pg/mL, *n* = 15) and 19.7% at high melatonin concentration (mean = 17.5 pg/mL, *n* = 16).

Lens transmittance: *In-vivo* measurements of lens transmittance were obtained before and after surgery by means of spot reflectometry. In short, a beam of white light entered the eyes of the subjects and the spectral composition of the reflected light was measured. The technique has been described in detail in previous work [22,23].

### 2.3. Data Analysis

For analysis all timing data were set to Central European Time (CET). Also, in summer, time is expressed as CET, not as DST.

The sleep-wake rhythm was characterized from actigraphy data by means of sleep analysis 5 software (Cambridge Neurotech Ltd, Cambridge, UK) together with sleep logs. The Actiwatch algorithm scores wake or sleep per minute as follows. If ai is the activity score in minute i, then Ai = ai + (ai – 1 + ai + 1)/5 is compared with a threshold value T = 40. When Ai > T the bin is scored as wake; if Ai<T the bin is scored as sleep. Sleep onset is operationally defined as the first episode after bedtime of 10 consecutive sleep minutes with no more than 1 bin of wake within that time. The time from bedtime till sleep onset is sleep latency. Sleep end is the last sleep minute before a 10-min consecutive period of wake from get up time. Sleep duration was calculated as the difference between sleep offset and sleep onset. Midsleep was used as phase marker [24,25]. We also calculated the average activity of the 10 most active hours (M10) and of the 5 least active hours (L5), which have been shown to be sensitive parameters to describe the rest-activity rhythm in the elderly [26,27], and sleep efficiency (percentage of time spent asleep while in bed). M10 and L5 are expressed as counts/hour.

A bimodal skewed baseline cosine function was fitted to the melatonin profiles before cataract surgery. This function allows for bimodality and skewness [28]. The maximum of an individual’s fitted curve before surgery was used to normalize the corresponding melatonin profile as well as the one obtained after cataract surgery. Dim light melatonin onset [29] and dim light melatonin offset were defined as the times at which 25% of the maximum value was crossed in the increasing and decreasing part of the fitted curve, respectively.

The phase angle differences (shown in decimal hours) were computed between the average sleep onset over the three weeks and both DLMO and DLMOff.

## 3. Results

### 3.1. Subjects

Fourteen participants (5 males and 9 females) between 66 and 87 years old (average age ± standard deviation (SD): 77.9 ± 5.2 years) participated in the study. All participants were retired. The average chronotype (MSF, based on MCTQ data) was: 3:25 h ± 58 min (SD). Two subjects ended the study with only one eye operated. In one case the subject was satisfied with the result, in the other there was a medical reason. After cataract surgery, 13 subjects had received Sensar implant lenses (UV absorbing hydrophobic acrylic) and one received a Tecnis CL implant lens (UV blocking SLM-2 Silicone) (Abbott Medical Optics inc., Santa Ana, CA, USA). Both lens types allow for short (blue) wavelength transmittance (as opposed to blue-blocking lenses). The single-eye operation subjects showed no deviating behaviours (*i.e.*, values within 2 SD from the average behaviour) in the parameters assessed in this study and were therefore included in the analysis. In comparison to pre-surgery, the transmittance of short wavelengths (between 420 and 500 nm) for all subjects was found to increase on average by a factor of 4.0 after cataract surgery [23].

The time of the year at which the measurements before and after cataract surgery were taken was not evenly distributed; by coincidence 10 subjects started during the summer months, in the presence of daylight saving time (DST) (before surgery measurements) and finished during the winter time (CET) months (after surgery measurements), while the other 4 subjects started in winter and finished during summer. It is conceivable that in addition to a possible effect of lens replacement, changes due to season (and DST) influenced the results [30].

### 3.2. Sleep-Wake Rhythms

**Figure 1:**
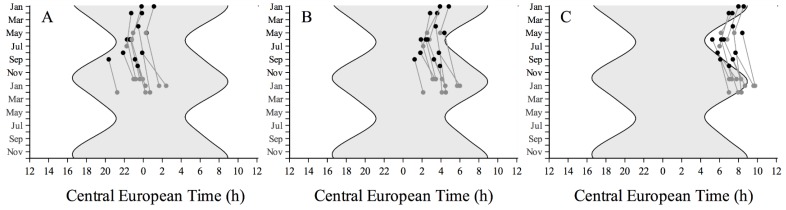
Sleep timing. Individual raw measurements of (**A**) sleep onset, (**B**) midsleep, and (**C**) sleep offset before (black circles) and after (grey circles) cataract surgery across the year as a function of Central European Time (CET). The grey area represents the night time.

Data on average sleep onset, midsleep, and sleep offset before and after surgery of each individual are shown in Figure 1. We observe a slight tendency for the sleep-wake rhythm to lock onto dawn rather than dusk, *i.e.*, both onset and end of sleep tended to be later in winter than in summer. Because conditions were not randomized across seasons, the comparison between before and after surgery is confounded with the seasonal change of phase, which itself is likely to be affected by the summer introduction of DST as shown by Kantermann and colleagues [30]. We used their data on changes in mid sleep on free days (MSF) across the year to correct our sleep timing data for the confounding effect of season. We first fitted a sine curve using CircWave version 1.4 (provided by Roelof Hut, University of Groningen [31]) through the midsleep data of Kantermann *et al.* and estimated for each day in the year a correction factor by calculating the deviation from the overall annual mean midsleep [30]. Secondly, we corrected each participant’s midsleep data by subtracting the appropriate correction factor for that date. The same procedure was followed for the other sleep timing parameters (sleep onset, offset).

**Figure 2:**
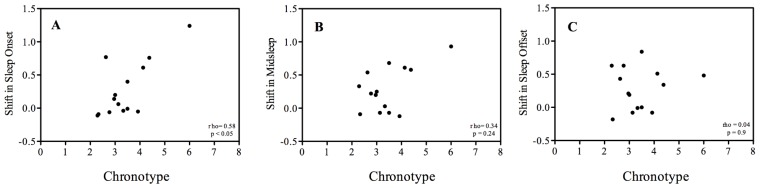
Correlations. Correlation (Spearman) among chronotype (MSF) and the difference between the before and after cataract surgery measurements of (**A**) sleep onset, (**B**) midsleep, and (**C**) sleep offset.

Sleep timing after correction for seasonal confounding effects is summarized in Table 1. A significant delay of 17 min was observed for midsleep after surgery compared to before surgery. Not all subjects responded in the same way. To explore these individual differences we assessed how the observed differences correlated with, on the one hand chronotype (MCTQ) and on the other with the improved lens transmittance factor (average factor: 4 ± 1.5 SD) as a result of cataract surgery [23]. A significant positive correlation was found between the observed shifts in sleep onset and chronotype; the later the chronotype the more sleep onset delayed after surgery (Spearman’s rho = 0.58, *p* < 0.05) (Figure 2A). The shift in midsleep and sleep offset did not show a significant correlation with chronotype (Figure 2B,C). No correlation was observed between the changes in the onset, midsleep, and offset of sleep, and the improvement factor in lens transmittance due to cataract surgery (Spearman’s rho = 0.12, 0.11, 0.09 for the onset, midpoint, and offset of sleep, respectively; all *p* > 0.7). Sleep duration was virtually the same before and after surgery and both the onset and offset of sleep were significantly delayed on average by about 17 min in response to cataract surgery. No significant (n.s.) differences were observed in the time it took the participants to fall asleep (sleep onset latency).

There are no data available in the literature of seasonal effects on aspects of the sleep-wake cycle other than timing. To estimate the effects of surgery on sleep efficiency, M10, and L5 the following approach was taken. We hypothesized that the effect of season contributed positively to the total effect of season + surgery in those subjects who started during the summer months while season was hypothesized to contribute negatively in those subjects starting in the winter. We assumed that the effect of cataract surgery was not dependent on season. The hypothesized relationship is as follows:

Group 1 (*n* = 4, summer-winter): average shift = effect of surgery + effect of season

Group 2 (*n* = 10, winter-summer): average shift = effect of surgery − effect of season

**Table 1:** Sleep timing outcomes obtained by means of a combination of sleep diaries and actiwatch data after subtracting seasonal-confounding effects (see description in Section 3.2). Data are shown as average ± SD. N = 14.

	Before Surgery	After Surgery	F	*p*	Difference
Sleep Onset (CET)	23:12 ± 1:06	23:28 ± 1:13	5.8	< 0.05	16 ± 25 min
Midsleep (CET)	03:10 ± 51 min	3:28 ± 59 min	10.3	< 0.01	17 ± 19 min
Sleep Offset (CET)	7:08 ± 49 min	7:25 ± 50 min	10.7	< 0.01	17 ± 10 min
Sleep Duration (CET)	7:56 ± 38 min	7:56 ± 40 min	0.006	0.94	0.5 ± 24 min
Sleep Onset Latency (min)	10.5 ± 6	13 ± 9.8	2.26	0.16	2.5 ± 6 min

With two equations and two unknown variables, by entering the average shifts of group 1 and group 2 in the formulas, we were able to estimate the average effect of surgery and of season separately. The average season effect is then used to correct individual data. The correction factors for season we obtained were: −0.49, 0.14, and −0.68 for sleep efficiency, M10, and L5, respectively. The size of the factor indicates a very small effect of season in these parameters. After correction of the individual data for this season factor, effects of cataract surgery were observed neither in sleep efficiency (average ± SD: before surgery: 81.3% ± 8.1% after surgery: 82.8% ± 8.2%, F = 1.27, n.s.), nor in M10 (average ± SD: before surgery: 53.4 ± 3.3 counts, after surgery: 53.6 ± 4.0 counts, F = 0.08, n.s.), nor in L5 (average ± SD: before surgery: 9.4 ± 4.9 counts, after surgery: 9.8 ± 5.6 counts, F = 0.91, n.s.).

### 3.3. Melatonin Rhythms

**Figure 3:**
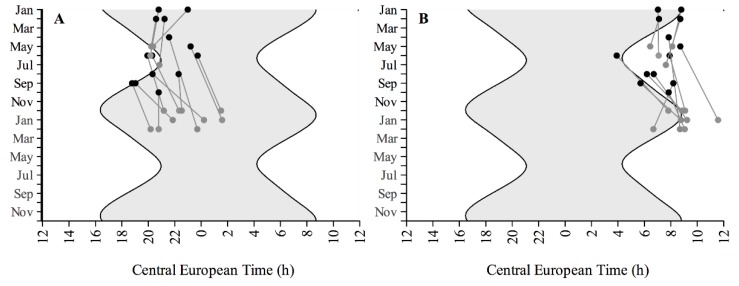
Timing of the melatonin rhythms. Individual raw measurements of (**A**) dim light melatonin onset; and (**B**) dim light melatonin offset before (black circles) and after (grey circles) cataract surgery across the year as a function of Central European Time (CET). The grey area represents the nights.

**Figure 4:**
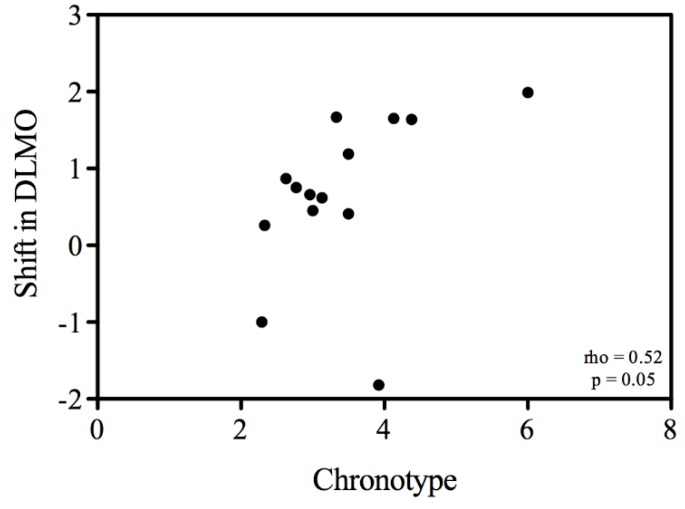
Correlation (Spearman) between chronotype (MSF) and the difference between the before and after cataract surgery measurements of dim light melatonin onset (DLMO).

DLMO and DLMOff before and after surgery with respect to CET are shown in Figure 3. In accordance with the sleep behaviour, we observe a tendency to lock onto dawn. DLMO and DLMOff tend to be later in winter than in summer. Considering MSF is a good phase marker for human subjects, we used the same seasonal information on the effects of midsleep and applied the same method of correction as we did for the sleep timing data to reveal the effects of surgery on DLMO and DLMOff (Table 2). DLMO was significantly delayed by about 40 min after surgery (F = 5.69, *p* < 0.05). Although the shift in melatonin rhythms (40 min) after surgery seems to be larger than the changes observed in the rhythms of sleep (about 17 min), this difference was not significant (t = −1.7, *p* = 0.1). Not all subjects showed the same degree of phase delay in DLMO. A positive correlation between the shift in DLMO after surgery and chronotype was on the verge of significance (Spearman’s rho = 0.52, *p* = 0.05) (Figure 4). The delay observed in DLMOff after surgery compared to before surgery (+47 min) was almost significant (F = 4.57, *p* = 0.05) and did not correlate with chronotype (Spearman’s rho = 0.16, *p* = 0.6). Neither shifts of DLMO nor of DLMOff correlated with the improvement factor in lens transmittance as a result of the cataract surgery (Spearman’s rho = 0.37; 0.26; *p* = 0.19; 0.37 for DLMO and DLMOff, respectively).

No significant differences were observed between the mean phase angle between DLMO and sleep onset before (−1.96 h; SD 1.47) and after (−1.56 h; SD 1.49) lens replacement (F = 3.05, *p* = 0.1). Phase angle between DLMOff and sleep onset was also not significantly different between before (8.14 h; SD 1.6) and after lens replacement (8.65 h; SD 0.95) (F = 1.89, *p* = 0.19). As expected from this observation, a significant and positive correlation was found between the observed shift in sleep onset in response to cataract surgery and the observed shift in DLMO in response to cataract surgery (Pearson’s r = 0.63, *p* < 0.05).

The change in phase angle between DLMO and sleep onset and between DLMOff and sleep onset as a consequence of cataract surgery did not significantly correlate with chronotype (Spearman’s rho = 0.46, *p* = 0.1 and Spearman’s rho = −0.88, *p* = 0.8, respectively). Phase angle difference due to cataract surgery did not correlate with the improvement factor in lens transmittance (Spearman’s rho = 0.36 *p* = 0.2 for phase angle between DLMO and sleep onset; Spearman’s rho = 0.1, *p* = 0.7 for the phase angle between DLMOff and sleep onset).

Estimations of the effect of season on the amplitude of the nocturnal melatonin rhythm were corrected in the same way we calculated the effects for sleep efficiency, L5, and M10. After subtracting the effect of season, we observed no significant changes in the absolute amplitude of the melatonin rhythm after cataract surgery (10.3 pg/mL; s.d. 7.4) as compared to before surgery (13.1 pg/mL; SD 10.6. F = 1.52, *p* = 0.24).

**Table 2:** Timing of dim light melatonin onset (DLMO) and offset (DLMOff) after subtracting seasonal confounding effects (see description in Section 3.2). Data are shown as average ± SD. N = 14 and 13 for DLMO and DLMOff, respectively.

	Before Surgery	After Surgery	F value	*p* Value	Difference
DLMO	21:14 h ± 1:30 h	21:54 h ± 1:41 h	5.69	< 0.05	40 min ± 1:03 h
DLMOff	07:20 h ± 1:12 h	8:07 h ± 1:08 h	4.57	0.05	46 min ± 1:19 h

## 4. Discussion

In recent years, several researchers have raised the question of possible effects of the aged lens and its replacement on the circadian system [23,32,33,34,35], on sleep [36,37,38,39,40] and melatonin rhythms [37,40] in particular. We set out to contribute to these questions by assessing objective measurements such as the timing of the sleep-wake rhythm and the nocturnal melatonin rhythm as well as sleep quality before and after cataract surgery within the same individuals.

Unfortunately, due to our uneven distribution across seasons of the before and after surgery measurements, corrections had to be applied to our data. Corrections were based on the seasonal effects on human sleep observed by Kantermann and colleagues [30]. Their data is mainly based on the German population, which is slightly earlier than the Dutch one [41]. In view of the sample size and time resolution of their study, and the fact that we use differences and not actual values for our corrections, our approach remains the best possible solution. After removing the confounding effects of seasons (which included the annual shift to Daylight Savings Time), our study suggests that the implantation of new transparent lenses after cataract surgery leads, on average, to a delay of the sleep-wakefulness and nocturnal melatonin rhythms. This was unexpected in view of the relative increase in Zeitgeber strength after cataract surgery that is supposed to lead to a phase advance [24]. Nonetheless, it is consistent with the later phase observed in the young (clear lens) population as compared to the elderly one (yellowish lens) [41,42,43,44]. The mechanism behind the advanced sleep-wake cycle that is observed in the elderly is not known [44,45,46,47]. It may be hypothesized that the aging of the lens contributes to it. Exposure to relatively larger amounts of especially blue light in the evening in comparison with the situation before surgery could cause the observed phase delay. During the evening hours, when light intensities are low compared to daytime, an increase in photons by a factor 4 (~0.5 log unit) could be sufficient to raise light levels just above a critical value to induce a shift in phase [48,49] and counteract the advancing effects of morning light. Moreover, the observation that phase advances, and not delays, are attenuated in the elderly could enhance this delaying effect [14]. The shift to a later phase may also be explained by a reduction in sleep pressure. If subjects go to bed later due to the activating effects of light [50], this would lead to a later waking up time and consequently to a shift in the exposure to the light-dark cycle. Complementary to this line of thought, the fact that vision has been enhanced after surgery may have allowed participants to engage more in activities such as reading or watching TV, hence staying up later in the evening hours and consequently exposing themselves to more minutes of evening light. Distinguishing between these different mechanisms is challenging. While a positive correlation was found between the changes we observed in sleep rhythms and the ones observed in the melatonin rhythms after surgery, it is not possible to define a causal relationship between more minutes of evening light as consequence of a delayed sleep, and the shift in the rhythm of melatonin. Moreover, the fact that DLMO shows a rather, though not significant, larger shift (40 min) compared to the shift in sleep onset (about17 min) supports the idea that it is not only activity in the evening delaying sleep and because of that delaying melatonin, but that also the phase delaying effect of light on the clock plays a role. Otherwise, the opposite tendency might have been expected. The positive correlation between chronotype and phase shifting effects that we observed in our data further supports that evening light might be playing an important role; later chronotypes are exposed to more light in the evening allowing, possibly, for larger delays as compared to earlier chronotypes. The present correlations should be taken with caution since a single individual, the latest chronotype in this group, mainly drives the observation. We are however not in a position to remove this individual from the database since we have not used sleep characteristics as an in- or exclusion criterion and this chronotype value is not an uncommon value in other groups. Given the variability of chronotypes in the population, this remains a valid point for discussion. As a matter of fact, previous studies have shown that both in young subjects [51] and in the elderly [52], early chronotypes are exposed to relatively more light earlier in the day than later chronotypes who are more exposed to light later in the day. Correlations between chronotype and phase shifting effects have previously been shown in the elderly. Benloucif *et al.* (2006) reported that the phase delay in the melatonin rhythms of elderly subjects correlated with the offset time of sleep [53]. Although sleep occurs at a later clock time after lens replacement, the lack of a change in phase angle difference between sleep onset and DLMO before and after surgery shows that sleep occurs at a similar circadian time as before surgery. This could partially explain why we do not observe changes in the efficiency of the sleep (see below). The lack of correlation with the improvement factor in lens transmittance after surgery suggests that the observed changes are due to the relative overall increase in light input (due to an increase in lens transmittance after surgery) and not to the specific improvement factor per participant and their respective changes in sleep-wake and melatonin rhythms. Tanaka and colleagues (2010) recently reported no differences in the timing of sleep or of the melatonin rhythms between before and after cataract surgery [37]. Given the inter-individual variation in timing observed in their participants, it would be interesting to consider taking chronotype into account in the analysis. A more recent study showed an approximately 22 min advance of the most active 10-h interval and no changes in the timing of the melatonin rhythms after surgery of one eye [40]. In contrast to our study, not only did they observe an advance but also they only found a difference at the activity level that is not reflected in the melatonin rhythms. Whether the lack of detection of a shift in the melatonin rhythms is due to its sampling rate (every 4 h) remains to be seen. Alternatively, only an effect at the level of behavior was observed and not per se a change at the level of the clock. In line with our observations on seasonal effects, the authors also mention the relevance of considering the seasonal aspect when assessing long term follow-ups.

We did not observe systematic differences in the parameters related to the amplitude of the circadian system (*i.e.*, L5, M10, sleep efficiency, and melatonin amplitude). Unfortunately, there is no literature-based information to correct our data for the uneven distribution across seasons of the measurements before and after lens replacement. We used our own records to estimate this effect, and after correction we observed no differences due to lens replacement. Asplund and Lindblad studied subjective sleep quality (*i.e.*, poor sleep, frequent awakenings, and difficulty in falling asleep after nocturnal awakening) one and nine months after lens replacement in Norway [36,54]. The authors did not quantify sleep quality but instead gave the proportion of subjects who reported an increase in sleep quality. From a database of 407 patients (35% male), they report a decrease in the proportions for poor sleep from one month to nine months after cataract surgery going from ~28% to ~16% in males and from ~37% to ~31% in females. Time of year was not taken into account in the analysis of Asplund and Lindblad, and it is conceivable that, especially at high latitudes, changes in environment at different times of the year may have had an impact on sleep quality [55,56]. Recent reports on the Pittsburgh Sleep Quality Index (PSQI) showed both a short-term (it disappears in the 12 months after surgery measurement) improvement [39] as well as no changes [40] after surgery. In a recent study we simulated the aging of the ocular lens in young subjects by means of orange soft contact lenses. We observed an increase in sensitivity to light (measured as melatonin suppression) after two weeks of reduced short wavelength input, which could be explained by adaptation [57]. This was also reflected in the lack of changes in either sleep or melatonin rhythms between conditions. Najjar *et al.* (2014) show that the increased lens filtering that occurs with aging does not lead to a proportional change in the response of the NIF [58]. Compensatory mechanisms (e.g., adaptation) might take place in healthy elderly as suggested by the authors. Their comparisons are between (young *vs.* elderly) and not a within (elderly with and without blue input) nature. In the elderly, not only the lens is aged but the whole system as well. For instance, at the level of the retina, structural changes occur; the number of photoreceptors decline [18,19,20]. There is also evidence for neural desynchrony in the SCN of aged mice [59]. All these factors could lead to a less robust circadian system that becomes more responsive to changes that the young might be filtering out. How adaptation might be influencing the different responses reported so far is not clear yet.

## 5. Conclusions

Light is an important Zeitgeber for human entrainment, and the reduction of light input through the aged eye has often been hypothesized to be causal in circadian misalignment and sleeping problems in the elderly. The present study intended to provide objective observations on the consequences of (bilateral) cataractous lens replacement, resulting in an increase of light input through the eye, for circadian sleep-wake and melatonin rhythms. The results indicate that this increase in light input through the eye of aged people may, to some extent, normalize the advanced phase of sleep and melatonin that are often observed in elderly. To what extent this delay is due to a relatively larger increase in evening light exposure as compared to morning (advancing) light exposure, either through more light entering the eye, and/or as exposure to more minutes of evening light due to behavioral changes needs further research. In addition we promote awareness in considering possible seasonal influences when conducting a longitudinal study, as well as taking chronotypes into account. A wide range of responses of the NIF system after replacement of cataractous lenses have been reported. Both aspects may be important factors that could impact the effects of lens replacement and entrainment in human subjects.
